# Perioperative Outcome in Dogs Undergoing Emergency Abdominal Surgery: A Retrospective Study on 82 Cases (2018–2020)

**DOI:** 10.3390/vetsci8100209

**Published:** 2021-09-28

**Authors:** Aida Fages, Carme Soler, Nuria Fernández-Salesa, Giuseppe Conte, Massimiliano Degani, Angela Briganti

**Affiliations:** 1 Department of Veterinary Sciences, Veterinary Teaching Hospital “Mario Modenato”, University of Pisa, 56122 Pisa, Italy; massidegani@gmail.com (M.D.); angela.briganti@unipi.it (A.B.); 2 Veterinary Teaching Hospital, Catholic University of Valencia “San Vicente Mártir”, UCV, 46018 Valencia, Spain; mdc.soler@ucv.es (C.S.); Nuria.fernandez@ucv.es (N.F.-S.); 3 Small Animal Medicine and Surgery Department, Catholic University of Valencia “San Vicente Mártir”, UCV, 46018 Valencia, Spain; 4 Department of Agriculture, Food and Environment, University of Pisa, 56100 Pisa, Italy; giuseppe.conte@unipi.it

**Keywords:** emergency, laparotomy, perioperative complications, dogs

## Abstract

Emergency abdominal surgery carries high morbidity and mortality rates in human medicine; however, there is less evidence characterising the outcome of these surgeries as a single group in dogs. The aim of the study was to characterise the clinical course, associated complications and outcome of dogs undergoing emergency abdominal surgery. A retrospective study was conducted. Dogs undergoing emergency laparotomy were included in the study. Logistic regression analysis was performed to identify variables correlated with death and complications. Eighty-two dogs were included in the study. The most common reason for surgery was a gastrointestinal foreign body. Overall, the 15-day mortality rate was 20.7% (17/82). The median (range) length of hospitalisation was 3 (0.5–15) days. Of the 82 patients, 24 (29.3%) developed major complications and 66 (80.5%) developed minor complications. Perioperative factors significantly associated with death included tachycardia (*p* < 0.001), hypothermia (*p* < 0.001), lactate acidosis (*p* < 0.001), shock index > 1 (*p* < 0.001), leukopenia (*p* < 0.001) and thrombocytopenia (*p* < 0.001) at admission, as well as intraoperative hypotension (*p* < 0.001) and perioperative use of blood products (*p* < 0.001). The results of this study suggest that mortality and morbidity rates after emergency abdominal surgery in dogs are high.

## 1. Introduction

Emergency abdominal surgery is a common procedure in both veterinary and human medicine. In human medicine, emergency surgery accounts for 11–15% of all total surgeries; however, it is responsible for almost 50% of all postoperative deaths and 30% of all surgical complications [1].

Emergency abdominal surgery carries an overall high risk of mortality, with rates of 11–23% commonly reported in human medicine [2,3,4]. Causes for these high mortality rates are multifactorial and different risk factors for death have been reported, including age [1,2,3,5,6], American Society of Anaesthesiology (ASA) classification [2,3,5,6,7], preoperative comorbidities [8], presence of sepsis, disseminated cancer [1,2] and development of complications [6].

Emergency surgery is also a known risk factor for anaesthetic-related death in small and large animals [8,9].

The term emergency abdominal surgery includes a wide variety of surgical procedures performed to treat potentially life-threatening conditions, for which the presentation of the patient, perioperative management and the severity of the process can vary significantly. These surgical procedures are performed a short time after presentation, without anticipated planning, and the period for stabilising these patients is limited, due to the urgent need to perform the surgery as soon as possible. Moreover, these surgeries are usually performed outside of normal working hours, with reduced staff and higher personnel fatigue when compared with scheduled surgeries. All these factors are likely to contribute to the high risk of these patients and the poorer outcomes when compared with general non-emergency surgery [1,10].

Enhanced recovery after surgery (ERAS) protocols have been largely applied in human medicine to reduce morbidity and mortality of several surgical procedures [11,12]. ERAS protocols highlight the importance of establishing a specific pathway for the patients focusing on preoperative, intraoperative and postoperative components which comprehend a multidisciplinary approach (emergency medicine, surgery, anaesthesia, diagnostic, intensive care, internal medicine). Reports of use of these ERAS protocols in emergency veterinary surgery are absent but the first indication to build a specific ERAS protocol is to individuate the critical points of the procedure in order to act on them to ameliorate the outcome [13]. 

The first aim of our study was to report signalment, diagnosis, clinical course, complications and outcome during the perioperative period of a cohort of dogs undergoing emergency abdominal surgery. The second aim was to identify factors related to death and complications in this population of dogs. We believe this information could be of help in designing future enhanced recovery programs in this potentially vulnerable patient group.

## 2. Materials and Methods

### 2.1. Study Design

This was a retrospective observational study conducted in two institutions (the University of Pisa “Mario Modenato” Veterinary Teaching Hospital and the Catholic University of Valencia (UCV) “San Vicente Mártir” Veterinary Teaching Hospital). 

### 2.2. Data Collection

Medicals records for all dogs undergoing emergency surgery from January 2018 to March 2020 were reviewed. Dogs that underwent emergency open abdominal surgery and have complete medical records were included in the study. Emergency surgery was defined as surgery that was not planned the day before. Caesarean surgeries were excluded from the study. Multiple surgeries involving different anaesthetic episodes in the same dog related to the first surgery were treated as a complication of the first surgery. Multiple surgeries involving the same dog at different periods of time were treated as different cases.

Data collected from medical records included breed, sex, age, body weight, body condition score (BCS) (measured by a 5-point scale 1 to 5, 1: emaciated, 2: lean, 3: ideal, 4: overweight and 5: obese), presence of preoperative comorbidities, clinical signs at admission, physical examination findings, shock index (SI) value (heart rate (HR)/systolic arterial pressure (SAP)), presence of septic peritonitis at admission and preoperative and postoperative clinical laboratory findings (haemato-biochemical and blood gas analysis). Preoperative comorbidities were retrospectively classified using a modification of the Charlson Comorbidity Index score used in human medicine [14] (Table 1). The criteria for diagnosis of septic peritonitis at admission were the evidence of peritoneal effusion (with abdominal ultrasound or at the time of surgery) and the identification of intracellular bacteria and/or positive bacterial culture results for a peritoneal fluid sample. 

Other data recorded included indication for surgery, length of anaesthesia, length of surgery, requirement of blood product transfusions, use of vasopressors, use of synthetic colloids, complications, duration of hospitalisation and 15-day mortality. 

Complications were divided into intraoperative and postoperative. Intraoperative complications evaluated were hypotension (mean arterial blood pressure < 60 mm Hg as measured via a direct or indirect method), tachycardia (heart rate > 130 bpm), bradycardia (heart rate < 50 bpm) and hypothermia (rectal temperature < 36 °C). Postoperative complications were divided into minor and major (Table 2). Intraoperative blood pressure measurements were considered valid for the study if obtained with an invasive method: a pressure transducer was connected to a non-distensible specific line filled with heparinised solution (5 UI/mL) to a dorsal pedal artery of the dog. The system was zeroed at the level of the right atrium before starting the measurements. For the pre and postoperative monitoring, oscillometric or Doppler methods were used, paying attention to the dimension (height) of the cuff (about 40% of the circumference of the limb).

### 2.3. Statistical Analyses

For the statistical analysis, some perioperative variables were retrospectively divided in categories on the basis of the age (young < 2 years; adult 2–8 years and old > 8 years) [15], BCS (≤2; 3 and ≥4), weight (<10; 10–25 and >25 kg), comorbidity index (CI) at admission (0; 1 and ≥2), SI at admission (SI ≤ 1 and SI >1), temperature at admission (<37.5; 37.5–39.1 and >39.1 °C), pH value at admission (<7.3; 7.3–7.45 and >7.45), lactate value at admission (<2.5; 2.5–4.5 and >4.5 mmol/L), platelet count at admission (<175; 175–500 and >500 × 103/μL) haemoglobin value at admission (<13; 13–21 and >21 g/dL), white blood cell (WBC) count at admission (<6; 6–16.9 and >16.9 × 103/μL) and duration of surgery (<70; 70–120; >120 min [16]).

Descriptive statistics were calculated and expressed as percentages. Data were analysed for distribution with a D’Agostino–Pearson test. Parametrical normally distributed data were expressed as mean and standard deviation (SD); non parametrical and not normally distributed variables were expressed as median (range). A Student *t*-test or a Mann–Whitney test were used to compare data between survivors and non-survivors. Statistical significance was set at *p* < 0.05. A logistic regression model was used to evaluate univariable association of perioperative variables with mortality and postoperative complications. Model fit was evaluated using the Hosmer Lemeshow goodness-of-fit test statistic and the area under the ROC curve (Hosmer and Lemeshow, 2000; Dohoo and Stryhn, 2009). Results are reported as odds ratio (OR). Statistical significance was set at *p* < 0.05. Prism Version 6.0 (GraphPad Software Inc., San Diego, CA, USA) and JMP software (SAS Institute Inc., Cary, NC, USA) were used to develop all statistical procedures.

## 3. Results

The initial medical record search identified 142 possible cases of emergency abdominal surgery in dogs. All but 92 were removed because of incomplete medical records. An additional 10 dogs that underwent caesareans sections were not included. Eighty-two dogs met the inclusion criteria during the study period. Of the 82 dogs enrolled, 45 (55%) were females (35 sexually intact and 10 spayed) and 37 (45%) were males (31 sexually intact and six castrated). The median age was 7.1 (0.4–15.6) years and the median body weight was 18.5 (1.7–50) kg. Thirty breeds were included in the study. The most common breeds were mixed-breed (*n* = 23; 28%), Boxer (6; 7.3%), German Shepherd (6; 7.3%), Yorkshire Terrier (5; 6.1%), Labrador Retriever (3; 3.6%), Weimaraner (3; 3.6%), Jack Russell Terrier (3; 3.6%), Dachshund (3; 3.6%) and Cocker (3; 3.6%). On the basis of a body condition score scale, 17/82 (21%) were considered thin or cachectic (BCS ≤ 2) and 11/82 (14%) were considered overweight or obese (BCS ≥ 4). 

The most common clinical signs at presentation were lethargy (43; 52%), vomiting (36; 44%), anorexia (33; 40%), diarrhoea (19; 23%) and distended abdomen (15; 18%). Tachycardia (HR > 140 bpm for small-breed dogs; HR > 100 bpm for large-breed dogs) was documented in 48 dogs (58%) and no cases of bradycardia were documented. Most dogs, *n* = 47 (57.3%), were normothermic, 17 (20.7%) were hyperthermic and 13 (15.8%) were hypothermic. Hypotension (SAP < 90 mm Hg) and tachypnoea were present in 18 (22%) and 31 (37.8%) dogs, respectively. 

Physical examination and laboratory findings recorded upon arrival of all animals and the differences between survivors and non-survivors are expressed in Table 3 and Table 4, respectively. Perioperative variables significantly associated with mortality are represented in Table 5.

The most frequent reason for surgery was a non-linear gastrointestinal foreign body (*n* = 31; 39%), followed by gastric dilatation and volvulus (GDV) (15; 18.3%), pyometra (15; 18.3%) and hemoperitoneum (8; 6.5%). Rare indications for surgery were linear foreign body (three), urolithiasis with uroperitoneum (two), dehiscence at the site of previous gastrointestinal incisions (one), gastric necrosis (one), diaphragmatic rupture (one), intestinal obstruction caused by adherences of previous surgery (one), intussusception (one), prostatic abscess rupture (one), postoperative septic peritonitis after sternotomy for pleural foreign body (one) and penetrating abdominal bite wound (one). Diagnosis was not correlated with outcome. Eleven out of eighty-two (13%) dogs had septic peritonitis. The presence of septic peritonitis at admission was not associated with failure to survive.

The median (range) durations of surgery and anaesthesia were 90 (30–230) and 150 (60–350) min, respectively. Colloids were administered to 25 (30.5%) patients and locoregional anaesthesia was performed in 29 (35.4%) patients. The intraoperative complications reported were tachycardia (51; 62.2%), hypotension (50; 61%), hypothermia (47; 57.3%) and bradycardia (7; 8.5%). Development of intraoperative hypotension was associated with mortality (*p* < 0.001), postoperative development of SIRS/sepsis (*p* < 0.001), anorexia (*p* < 0.01) and postoperative hypotension requiring use of vasopressors (*p* > 0.001). No other intraoperative variable was associated with mortality or the development of postoperative complications.

Oesophagostomy and nasogastric feeding tubes were placed in 20/82 and 2/82 dogs, respectively, for management of the patients’ nutritional requirements after surgery, and 11/82 dogs had a central venous catheter placed. The decision of feeding tube or central venous catheter placement was determined by the clinician at the time of surgery. 

Of the 82 patients, 24 (29.3%) developed major complications while minor complications were reported in 66 (80.5%) patients. Major complications reported were systemic inflammatory response syndrome (SIRS) or sepsis development (12), pulmonary complications (4), acute renal injury (AKI) (4), neurological signs (3) and need for surgical reintervention (1). Of the pulmonary complications, two dogs had pulmonary thromboembolism confirmed at necropsy, one dog had pneumonia caused by aspiration of gastric content and one dog had pulmonary haemorrhage caused by coagulation abnormalities. The development of major complications was associated with mortality (*p* < 0.001).

The most commonly encountered postoperative minor complication was anorexia, documented in 41/82 (50%) dogs, followed by tachycardia (*n* = 25), diarrhoea (21), hypotension (18), reduced intestinal motility (17) (checked daily by ultrasonographic evaluation), melena (14), vomiting (12), fever (12), abdominal pain (12) and surgical site complications (5). Oesophagostomy tube infection was present in 4 of 20 (20%) dogs. Some alterations in the postoperative laboratory values were also considered as minor complications, as leucocytosis, present in 26 of 44 dogs with data available (59.1%), hypoalbuminemia (14; 51.8%), electrolyte imbalance (23; 36.5%), hyperlactatemia (14; 24.1%), anaemia (27; 15.8%), hypoglycaemia (7; 12.3%) and leukopenia (3; 6.8%). Rare postoperative complications reported were arrythmias (*n* = 4), pancreatitis (4), phlebitis (3), peripheric oedema (2) and jugular thrombosis associated with central venous catheter (1). Fourteen of eighty-two (17%) dogs had no complications reported. Risk factors significantly associated with the development of some postoperative complications are reported in Table 6.

Eighteen dogs in this study received a blood product transfusion during the perioperative period. Thirteen (15.85%) dogs received fresh-frozen plasma and five (6.1%) dogs received packed red blood cells (RBCs). Postoperative vasopressors were used in 15 (18.3%) dogs. The requirement of blood product transfusion significantly correlated with mortality (*p* < 0.001) and postoperative development of SIRS (*p* < 0.01) and hypotension requiring vasopressor therapy (*p* > 0.001). The postoperative use of vasopressors was correlated to death (*p* < 0.001) and postoperative development of diarrhoea (*p* < 0.01), SIRS (*p* < 0.001), anorexia (*p* < 0.05) and major electrolyte imbalance (*p* < 0.05).

Sixteen (19.5%) dogs died or were euthanised during the perioperative period. One dog died 7 days after discharge because of complications related to concomitant disease (hepatic insufficiency). Death occurred in one dog during surgery and in 13 after surgery (between 5–132 h). The remaining two dogs were euthanised because of complications arising after surgery. Necroscopy was not performed at the behest of the owners.

A total of 66 (80.5%) dogs survived to discharge from the hospital and a total of 65 (79.3%) were alive at the follow-up visit 2 weeks after surgery. The overall 15-day mortality rate was 20.7% and median (range) duration of hospital stay for the dogs that survived to discharge was 3 (0.5–15) days. 

The median (range) duration of the long term follow up was 90 (15–425) days for all dogs. One dog treated for gastric dilatation-volvulus underwent surgery 4 months after the first surgery because of intestinal obstruction created by adherence of the first surgery and was euthanised in the postoperative period because of complications related to concomitant diseases. One dog treated for intestinal foreign body was readmitted to the hospital 2 months after surgery in shock because another intestinal foreign body was causing obstruction and was euthanised. The remaining dogs were all alive at the time of the last follow up. 

## 4. Discussion

To our knowledge, the present study is the first in which factors associated with death and complications were evaluated in dogs that underwent emergency abdominal surgery.

The present study demonstrated that emergency laparotomy is a high-risk procedure, with an overall 15-day mortality rate of 20.7% and total complication rate of 80.5% with 24.4% of major complications. Factors associated with death included presence of CI ≥ 2; BCS ≤ 2; tachycardia, hypothermia, lactic acidosis, thrombocytopenia and leukopenia at admission; intraoperative hypotension; development of major complications; and perioperative use of blood product. Normal haemoglobin value at admission was positively associated with survival to discharge.

In human medicine, the most common procedures performed in the emergency setting are intestinal surgeries [2,6]. We found similar results, but, whereas the most common indication for humans is intestinal obstruction caused by adhesions or neoplasia [2,6], a gastrointestinal foreign body causing mechanical obstruction was the most common indication for surgery in the present study, which is in accordance with previous research on gastrointestinal emergencies in dogs [17,18]. 

The documented overall mortality rate in human medicine for patients undergoing emergency abdominal surgery varies from 11% to 23% [2,3,4,5,6,7] and can be as high as 35–45% in the elderly population [6]. The 15-day mortality rate in this study was 20.7%. There is no comparable veterinary study with which to compare data; however, mortality rates reported in veterinary medicine for the most common surgical procedures encountered here, such as gastrointestinal, hemoperitoneum or GDV syndrome surgeries, range from 0.8% to 28% [18,19,20,21,22,23], 12% to 36% [24,25] and 12% to 26% [26,27,28,29,30,31,32,33], respectively.

Considering preoperative patient factors and their associations with mortality, the odds of non-survival increased with increasing CI and decreasing BCS. Previous research in people has found associations between malnutrition and presence of comorbidities with increasing postoperative complications and death [34]. It is also worth noting that most patients with CI ≥ 2 in the present study had a diagnosis of chronic enteropathy, and a relationship between high CI and low BCS that was not statistically evaluated cannot be excluded.

On the other hand, it is well established in people that mortality after emergency surgery increases with age [2,3,5]. This association was not found in the present study. This difference may be explained by differences in range of age between humans and dogs. Moreover, in contrast with studies in people, the most common reason for surgery in the present study was a gastrointestinal foreign body causing mechanical obstruction, condition commonly reported in young dogs [21,22,23].

Clinical signs in emergency abdominal surgery can vary significantly depending on the underlying condition. Most of these patients are in shock and require immediate treatment. In the present study, tachycardia, hypothermia and SI > 1 at admission were found to be associated with death. Tachycardia is defined as a heart rate that is more rapid than is appropriate [35] and from the literature, the range heart rate for tachycardia varies from 100 to 160 bpm depending on the size of the animals [35,36,37]. In this study, tachycardia was considered as heart rate higher 140 bpm; this could be a limit because for small animals, 140 bpm of heart rate may not be a real tachycardia, and thus, the relation between heart rate and blood pressure is fundamental to verify the critical patient status. These alterations can reflect decreased perfusion and the sympathetic compensatory response in patients with shock [38]. Hypothermia can be caused by decreased systemic perfusion and it is associated with reduced venous return [28]; tachycardia can occur for multiple reasons, such as pain and/or compromised cardiovascular state. Their association with mortality may be related to the severity and duration of hypoperfusion at presentation. This is in accordance with other studies; tachycardia and hypothermia have been identified as risk factors for death in dogs with hemoperitoneum [33] and GDV [28,39].

On the other hand, SAP values <90 mmHg at admission were not correlated with failure to survive. This could indicate that most patients arrived in a compensatory shock stage and increased HR at this stage is more accurate in predicting the severity of illness than SAP. Moreover, the method of SAP measurement (oscillometer or doppler) was not standardised, and that could have altered the results. On the other hand, median (range) SI value of survivors (0.94; 0.3–2.8) was significantly (*p* = 0.01) lower than value of non-survivors (1.2; 0.6–3.3), and SI > 1 was significant in predicting mortality in the multiple regression analysis. This is in accordance with previous research that support that SI is more accurate in predicting mortality in comparison with low SAP values [40], and SI has been associated with shock and mortality in both humans [41] and dogs [42]. These results point out the need for haemodynamic stabilisation before surgery for these patients and that the SI, a powerful and easily calculated tool, should always be taken into account to predict more adequately the shock status of the patient.

Dogs that failed to survive had a median (range) plasma lactate concentration of 5 (0.6–16.6) mmol/L and a median venous pH of 7.23 (6.81–7.46) compared with a median plasma lactate concentration of 1.9 (0.3–14.8) mmol/L and a median venous pH of 7.39 (7.12–7.55) in survivors. Lactate > 4.5 mmol/L and pH < 7.3 were associated with death in the multiple regression logistic model. Lactic acidosis is a common finding in critically ill patients and is used as a marker of patient perfusion and resuscitation response both in human and veterinary medicine [38,43]. High serum lactate concentrations at admission have been associated with failure to survive in dogs after gastrointestinal surgery [18], secondary septic peritonitis [44], GDV [27,31,32,33] and trauma [45]. Nevertheless, the response of the lactate to intervention (fluid resuscitation) has been shown to provide a more accurate assessment of patient prognosis in veterinary [31,46] studies. In fact, in the present study, 10/82 dogs had a lactate value at admission above 6 mmol/L, and 5/10 (50%) survived. Lactate clearance was not evaluated in the present study, so we are not able to say if it had any influence on survival rate. Even so, patients with high plasma lactate concentration at hospital admission should be aggressively treated and closely monitored perioperatively to improve outcomes.

The presence of leukopenia and thrombocytopenia at admission were identified as risk factors for mortality (*p* < 0.001). WBC counts < 4.5 × 103/μL have been correlated with mortality in emergency abdominal surgery in human medicine [2]. The presence of leukopenia before surgery certainly evidences an immune imbalance or a severe inflammatory response and makes the dog more susceptible to any type of infection [47]. Thrombocytopenia may provoke perioperative severe blood loss. Fresh frozen plasma or platelets should be administered before surgery in severely leukopenic and thrombocytopenic patients.

Albumin value < 2.5 g/dL at admission was also associated with mortality (*p* < 0.001) and perioperative use of blood products (*p* < 0.001). Hypoalbuminemia is often associated with critical illness and can result in complications such as pulmonary oedema, enteral feeding intolerance, poor wound healing and hypercoagulability [48]. Hypoalbuminemia has been shown to be associated with increased morbidity and mortality in dogs [49,50,51]. Albumin concentration serves as a marker for disease and nutritional status and is essential for maintaining oncotic pressure [48]. It is worth noting that 36% of dogs that were hypoalbuminemic at admission developed postoperative hypotension, and all dogs that developed postoperative hypotension and had postoperative albumin values measured had postoperative hypoalbuminemia. The use of albumin supplementation has been shown to improve outcomes in dogs [50,51] and its use in these vulnerable patients should be considered.

Intraoperative hypotension was diagnosed in 50 (60%) of the dogs in the present study and was significantly associated with postoperative development of hypotension, SIRS/sepsis and anorexia; use of vasopressors in the postoperative period; perioperative use of blood products; and death. Similarly, Beck et al. [26] found that hypotension detected at any time during the hospitalisation period was associated with increased risk of death in patients with GDV. These results likely reflect the fact that dogs that developed intraoperative hypotension and required vasopressor therapy or blood product transfusions had more severe disease in general and were more likely to develop postoperative complications and fail to survive than other dogs. However, intraoperative hypotension episodes produce low tissue oxygenation that could certainly have worsened some conditions, such as SIRS or anorexia. A relationship between intraoperative hypotension and the development of postoperative anastomotic leakage after intestinal surgery has been previously demonstrated, and it has been attributed to local ischemia at the anastomosis site [19,20]. It could be hypothesised that intraoperative hypotensive episodes decrease enteric perfusion and enteric motility, which might affect postoperative ileus and, indirectly, postoperative anorexia development. 

Moreover, absolute and relative intraoperative hypotension has been found to be responsible for acute myocardial and renal injuries in human medicine [52]. Unfortunately, we did not evaluate the incidence of acute myocardial injuries because of lack of specific data, so we cannot draw any conclusions concerning this issue. Nevertheless, intraoperative normotension should represent an important therapeutic target to maximise clinical success, and invasive blood pressure monitoring is advised for these patients.

Postoperative major complications were encountered in 24.4% of dogs. This is also in accordance with human medicine, where major complications rates range from 33% to 47% [4]. Respiratory complications have been found to be associated with failure to discharge in dogs with hemoperitoneum [25]. In the present study, six dogs developed pulmonary complications, two of them pulmonary thromboembolic disease diagnosed at necropsy. Pulmonary complications were not individually analysed for associations with mortality due to the low number of cases, but the development of major complications after surgery was associated with mortality (*p* < 0.001), as has been found in human medicine [6]. In human medicine, it has also been demonstrated that the development of postoperative complications not only increases mortality in the immediate risk period, but also worsens long-term mortality [53].

Hypotension requiring use of vasopressor therapy was the most common postoperative major complication, present in 15 (18%) of the dogs in the present study. Of those 15 dogs, 10 (67%) failed to survive. The need of vasopressors, due to a hypotensive status, in the postoperative period was associated with death and postoperative development of SIRS, anorexia, diarrhoea and major electrolyte imbalance. In total, hypotension was documented in 18/82 (22%) of patients in the postoperative period. As mentioned above, hypotension is a severe hemodynamic disturbance that causes poor tissue perfusion, resulting in cellular suffering and potentially multi-organ dysfunction [26]. Frequent blood pressure monitoring measurements are mandatory in the postoperative period, and a constant invasive blood pressure monitoring should be considered in critical patients. 

In the postoperative period, SIRS was diagnosed in 12/82 (15%) dogs. Development of SIRS was associated with BCS ≤ 2; CI ≥ 2; tachycardia, SI > 1 and WBC < 6 × 109/L at admission; development of intraoperative hypotension; perioperative use of blood products; and postoperative use of vasopressors. Of the 12 dogs that developed SIRS in the postoperative period, 10 failed to survive. The development of SIRS in the postoperative period can be considered as worsening the patient’s recovery and assessing the evolution of the SIRS score daily in every patient should be considered. Unfortunately, one of the limitations of the study is that the SIRS score changes were not evaluated.

Minor complications were present in 80.5% of dogs in this study. The most common complication was anorexia, present in 41 (50%) of dogs. Anorexia was significantly correlated with age: adult and old dogs were 3.7 and 13.7 times, respectively, more prone to develop anorexia in comparison to young dogs (<2 years). In human medicine, it is well known that increasing age is a risk factor for postoperative ileus [6]. A reduction in the intestinal motility was identified in 17 dogs and the high prevalence anorexia in elderly patients could be related to it. Unfortunately, due to the retrospective nature of the study, it was not possible to analyse the details of intestinal motility—further prospective studies with a specific evaluation of the intestinal motility are needed to clarify this aspect. Weight was also found to be related to the development of anorexia. Dogs weighing less than 10 kg were more likely to be anorexic compared with dogs >10 kg. Other risk factors for the development of anorexia found in the present study were tachycardia, SI > 1 and pH < 7.3 at admission, intraoperative hypotension and postoperative use of vasopressors. All the conditions seem to be related to the severity of systemic injury, and critically ill patients seems to be more likely to develop anorexia in the postoperative period. Moreover, an experimental study on the pathophysiology of the perioperative causes of ileus identified the increase in catecholamines blood concentration as responsible for ileus in dogs [54]; this could, in some way, explain the relationship of anorexia with the postoperative use of vasopressors.

Nutrition is essential during recovery from illness or surgery. Early nutritional support has been shown to improve outcomes and shorten recovery time [55]. Caution should be taken in dogs with known risk factors for the development of postoperative anorexia, and feedings tubes placed at the time of surgery could be a good option so enteral nutrition can be started as soon as possible after surgery in critically ill dogs. Feeding tubes were placed in 22/82 dogs in the present study.

Surgical site complications were present in 5 of 82 dogs (6%) and were significantly correlated with low BCS. Surgical site infections of 2.5% to 18.5% are reported in small animal surgery, with variations associated with the type of surgery [56,57,58,59,60]. Undernutrition, emergency surgery and prolonged surgical times have been described as risks factors for surgical site infections in veterinary medicine [58,59,61]. In contrast, surgical time did not affect the development of surgical site complications in this study. In fact, surgical time was not related with any complication in the multiple logistic regression analysis, however, the maximum surgical time was 230 min. It may be concluded that if the patient is stable, operating time is not a critical variable to be considered.

Postoperative diarrhoea was associated with postoperative use of vasopressors. It could be hypothesised that severe hypotension requiring vasopressors can be a contributor to intestinal ischemia development that may promote morphological and functional changes that enhance intestinal permeability and can manifest clinically with diarrhoea [62]. Diarrhoea in the postoperative period was also associated with high haemoglobin value at admission and low BCS. In fact, postoperative vomiting was also associated with high haemoglobin value at admission. This may be explained by the fact that high haemoglobin value was related to the presence of dehydration at admission, and dehydrated patients were more likely to develop these complications. 

Postoperative major electrolyte imbalance was associated with high CI and postoperative use of vasopressors. It is not surprising that patients that needed vasopressors for the maintenance of normal blood pressures were more predisposed to electrolyte disorders. The most common electrolyte derangement found in this study was hypokalaemia and the most common comorbidity encountered in patients classified with CI ≥2 was chronic enteropathy. Hypokalaemia has been found to be associated with gastrointestinal disease in previous research [63] and could be one of the reasons between the association of high CI and major electrolyte derangement in the present study. Nevertheless, electrolyte concentrations should be closely monitored in these patients.

Perioperative use of blood products was associated with postoperative development of SIRS, hypotension requiring vasopressor therapy, and death. We did not differ between different types of blood products, such as packed RBCs or fresh frozen plasma, so it is difficult to make any conclusion about these results. Moreover, we did not evaluate separately massive transfusion, a known risk factor for postoperative complications and death in previous research [28]. Further study is warranted to investigate the impact on prognosis and needs for transfusions in dogs undergoing emergency abdominal surgery.

The median hospitalisation time was relatively short (3 days; range 0.5–15 days). It would appear that even if these patients usually require intensive monitoring, the median length of hospital stay is short.

This study has a number of limitations, the most important of which are the small sample size and the retrospective design, as collection and interpretation of the data may be subject to information bias. Due to the retrospective and multicentric nature of the study, the laboratory test to be performed was not standardised and there are a lack of specific data, such as postoperative albumin value in some patients. Types of treatment and resuscitation of the animals could have been different between clinicians of the different hospitals and may have distorted the outcome and clouded the findings. Surgeries were also performed by multiple surgeons with different degrees of experience, which could have affected the results and the development of some complications. Larger multicentre prospective studies evaluating outcomes in dogs undergoing emergency abdominal surgery should be explored.

## 5. Conclusions

In conclusion, mortality and morbidity rates after emergency abdominal surgery in dogs from this study are comparable to those reported in human medicine. Several variables that correlated with failure to survive and the development of postoperative complications were identified in our patient population. We believe this study provides useful information for the recognition of dogs at higher risk than others before emergency abdominal surgery and that may help clinicians identify patients that can benefit from more intensive monitoring and intervention, as well as to inform owners about prognosis. This information could be used to create specific ERAS protocols for optimising recovery after surgery in this dog population. Stabilising these parameters before surgery and verifying the stabilisation of the patient through end points, providing intensive intraoperative and postoperative monitoring, and preventing some conditions, such as postoperative anorexia, with the use of feeding tubes, could be useful tools to improve outcomes. Further investigation in prospective studies is warranted to fully address the role of some interventions in improving outcomes.

## Figures and Tables

**Table 1:** Charlson Comorbidity Index modified classification.

Comorbidity Index (CI)	
0	No comorbidity present
1	Any malignancy, compensated diabetes, mild cardiomyopathy
2	Moderate or severe cardiomyopathy, liver disease, renal insufficiency, decompensated diabetes
3	Metastatic tumour

**Table 2:** Postoperative complications classification.

Minor Complication	Major Complications
Anorexia (more than 24 h without voluntary eating) Vomiting/regurgitation Diarrhoea Melena/haematochezia Tachycardia (HR > 140 bpm for small-breed dogs/HR > 100 bpm for large-breed dog) Bradycardia (HR < 60 bpm) Tachypnoea (RR > 40 rpm) Incisional complications (seroma, dehiscence, infection) without requiring surgical intervention Phlebitis Hypotension (MAP < 60 mm Hg/SAP < 90 mmHg as measured via a direct or indirect method) not requiring vasopressor therapy Hypertension (SAP > 180 mmHg) Fever Abdominal pain (assessed using the Glasgow Composite Pain Score) Anaemia (haematocrit < 30%) Leucocytosis (WBC > 19 × 10^3^/μL)/leukopenia (WBC < 5 × 10^3^/μL) Hypoalbuminemia (albumin < 2.5 g/dL Hyperlactatemia (lactate > 2.5 mmol/L) Hypoglycaemia (glucose < 60 mg/dL) Major electrolyte imbalance (K^+^ < 3.3 mmol/L or > 5.2 mmol/L; Ca^+2^ < 1.1 mmol/L or > 1.6 mmol/L; Na^+^ > 165 mmol/L or < 125 mmol/L; Cl^−^ > 140 mmol/L or < 90 mmol/L)	Hypotension requiring vasopressor therapy Pulmonary complications: respiratory failure requiring oxygen supplementation/mechanical ventilation Neurological signs Intra-abdominal suture dehiscence (retrospectively deemed to have occurred if results of postoperative abdominocentesis were consistent with sepsis or dehiscence was confirmed during a subsequent surgery or at necropsy) Surgical reintervention Acute kidney injury (creatinine levels increase 0.3 mg/dL from the initial value or creatinine values higher than 1.6 mg/dL) SIRS/sepsis: ≥ 2 of the following criteria: Hypo- or hyperthermia (<37.8 or >39.4 °C), Tachycardia (HR > 140 bpm/min), tachypnoea (RR > 20 bpm/min), leukopenia or leucocytosis (WBC (<6.0 or >16.0 × 10^3^/μL)) or immature (band) neutrophils > 3%)

HR = heart rate; bpm = beats per minute; RR = respiratory rate; bpm = breaths per min; MAP = mean arterial pressure; SAP = systolic arterial pressure; WBC = white cell count; SIRS = systemic inflammatory response syndrome.

**Table 3:** Physical examination findings at arrival and their associations with death.

Variables	All Dogs (*n* = 82)	Survivors (*n* = 65)	Non-Survivors (*n* = 17)	*p* Value
Heart rate (beats/min)	138 (80–247)	130 (60–220)	160 (80–250)	0.001
Respiratory rate (breaths/min)	40 (16–150)	44 (16–150)	32 (20–80)	0.08
Systolic arterial blood pressure (mmHg)	130 (70–240)	130 (70–270)	130 (70–200)	0.65
Body temperature (°C)	38.4 (33.4–40.1)	38.6 (35.3–40.2)	37.6 (33.4–40.1)	0.002
Shock index (HR/SAP)	0.98 (0.6–2.85)	0.94 (0.3–2.8)	1.2 (0.6–3.3)	0.01

*p* values represent values for Student *t*-test or Mann–Whitney test (values of *p* < 0.05 were considered significant). Values are reported as median (range). Values significantly different between survivors and non-survivors are highlighted using bold characters. HR = heart rate; SAP = systolic arterial pressure.

**Table 4:** Laboratory values at admission of the complete cases and divided on the basis of the outcome (survivors and non-survivors).

Variables	All Dogs (*n* = 82)	Survivors (*n* = 65)	Non-Survivors (*n* = 17)	*p* Value
pH	7.38 (6.81–7.48)	7.39 (7.12–7.55)	7.23 (6.81–7.46)	<0.0001
PCO_2_ (mm Hg)	36.4 ± 9.4	35.2 ± 8.7	41 ± 10.8	0.03
BE (mmol/L)	−5.2 ± 6.1	−3.6 ± 4.5	−10.5 ± 7.9	<0.0001
HCO_3_^−^ (mm Hg)	19.7 ± 4.9	20.7 ± 4.2	16.2 ± 5.9	0.001
Glucose (mg/dL)	114 (5–356)	113 (30–274)	128 (5–356)	0.56
Lactate (mmol/L)	2.1 (0.3–16.6)	1.9 (0.3–14.8)	5 (0.6–16.6)	0.008
Na^+^ (mmol/L)	144 ± 7	144 ± 6	144 ± 8	0.96
K^+^ (mmol/L)	3.72 (2.9–5.1)	3.7 ± 0.4	4.3 ± 0.6	<0.0001
Cl^−^ (mmol/L)	113 (92–134)	111 ± 6	113 ± 8	0.35
Ca^+2^ (mmol/L)	1.31 (0.79–3.28)	1.23 (0.39–1.64)	1.28 (0.56–1.36)	0.83
WBC count (× 10^3^/μL)	12.92 (3.8–43)	13.2 (4.7–43)	10.5 (3.8–39)	0.17
Platelet count (× 10^3^/μL)	246 (8–590)	261 (40–590)	160 (8–484)	0.02
Haemoglobin (g/dL)	15.4 ± 4.5	15.7 ± 4	14.6 ± 6.1	0.38
Total proteins (g/dL)	6.6 ± 1.4	6.9 ± 1.4	5.3 ± 1.1	0.001
Creatinine (mg/dL)	0.9 (0.4–12.8)	0.9 (0.4–4.3)	1 (0.04–12.8)	0.88
Albumin (mg/dL)	3 ± 0.7	3.2 ± 0.7	2.1 ± 0.6	0.0002

**Table 5:** Variables correlated with death in the multiple logistic regression analysis.

Characteristics	*n* (%)	Mortality (%)	Odds Ratio	*p* Value
All patients	82	17/82 (21)		
BCS (scale 1–5)				
BCS ≤ 2	17 (21)	7/16 (43)	Base	*p* < 0.05
BCS 3	54 (66)	9/44 (20)	0.331	
BCS ≥ 4	11 (14)	0	<0.001	
CI (0–3)				
CI 0	58 (71)	10/58 (17)	Base	*p* < 0.001
CI1	16 (19)	0	0.830	
CI ≥ 2	8 (10)	7/8 (87.5)	33.600	
Tachycardia at admission				
Yes	48 (58.5)	16/48 (33)	Base	*p* < 0.001
No	34 (41.5)	1/34 (2.9)	16.498	
Temperature (°) at admission				
<37.5	13 (16)	8/13 (62)	Base	*p* < 0.001
37.5–39.2	47 (57)	7/47 (15)	0.109	
>39.2	17 (21)	2/17 (12)	0.083	
SI at admission				
>1	37 (45)	12/37 (32)	Base	*p* < 0.05
≤1	45 (55)	5/45 (11)	0.26	
pH value at admission				
<7.3	22 (27)	12/22 (55)	Base	*p* < 0.001
7.3–7.45	34 (41)	3/34 (9)	0.064	
>7.45	14 (17)	1/14 (7)	0.081	
Lactate (mmol/L) value at admission				
<2.5	43 (52)	6/43 (14)	Base	*p* < 0.001
2.5–4.5	11 (13)	0/11 (0)	0.860	
>4.5	17 (21)	11/17 (65)	11.305	
Platelet count (× 10^3^/μL) at admission				
<175	26 (32)	12/26 (46)	Base	*p* < 0.001
175–500	46 (56)	5/46 (11)	0.001	
>500	5 (6)	0/5 (0)	0.891	
WBC count (× 10^3^/μL) at admission				
<6	13 (16)	7/13 (54)	Base	*p* < 0.001
6–16.9	35 (43)	3/35 (8)	0.080	
>16.9	26 (32)	6/26 (26)	0.257	
Haemoglobin (g/dL) value at admission				
<13	23 (19)	8/23 (35)	Base	*p* < 0.05
13–21	52 (43)	5/52 (10)	0.199	
>21	7 (6)	3/7 (43)	1.406	
Albumin (g/dL) value at admission				
<2.5	11 (13)	6/11 (55)	Base	*p* < 0.001
2.5–4	34 (41)	2/34 (6)	<0.001	
>4	6 (7)	0/6 (0)	<0.001	
Perioperative blood product transfusion				
Yes	18 (22)	9/18 (50)	Base	*p* < 0.001
No	64 (78)	8/64 (13)	0.142	
Intraoperative hypotension				
Yes	50 (61)	17/50 (34)	Base	*p* < 0.001
No	32 (39)	0/32 (0)	<0.001	
Use of vasopressors in the postoperative period				
Yes	15 (18)	10/15 (67)	Base	*p* < 0.001
No	67 (82)	6/67 (9)	0.050	
Postoperative development of major complications				
Yes	20 (24)	14/20 (70)	Base	*p* < 0.001
No	62 (75)	3/62 (5)	171,128	
Postoperative development of minor complications				
Yes	67 (82)	16 (24)	Base	*p* < 0.001
No	15 (18)	0 (0)	23,008	

Variables significantly associated (*p* < 0.05) with mortality in the multiple regression analysis. The variables significantly associated with mortality are highlighted using bold characters. BCS = body condition score; CI = comorbidity index; SI = shock index; WBC = white blood cells.

**Table 6:** Risk factor analysis for postoperative complications.

Postoperative Complication	Variables	Odds Ratio	*p* Value
Anorexia	Age: >8 years	13.75	*p* < 0.001
Anorexia	Weight: <10 kg	0.195	*p* < 0.05
Anorexia	Tachycardia at admission	4.051	*p* < 0.01
Anorexia	pH < 7.3 at admission	0.246	*p* < 0.05
Anorexia	Intraoperative hypotension	0.241	*p* < 0.01
Anorexia	Postoperative use of vasopressors	0.208	*p* < 0.05
Vomiting	Hb value > 21 g/dL at admission	16.072	*p* < 0.01
Vomiting	Hypothermia at admission	0.228	*p* < 0.05
Diarrhoea	BCS ≤ 2	0.303	*p* < 0.01
Diarrhoea	Hb value > 21 g/dl at admission	8.000	*p* < 0.05
Diarrhoea	Postoperative use of vasopressors	0.148	*p* < 0.01
SIRS/sepsis	BCS ≤ 2	0.356	*p* < 0.05
SIRS/sepsis	CI ≥ 2	7.142	*p* < 0.05
SIRS/sepsis	Tachycardia at admission	18.157	*p* < 0.001
SIRS/sepsis	SI > 1 at admission	4.67	*p* < 0.05
SIRS/sepsis	Intraoperative hypotension	< 0.001	*p* < 0.001
SIRS/sepsis	Perioperative use of blood product	0.121	*p* < 0.01
SIRS/sepsis	Postoperative use of vasopressors	0.016	*p* < 0.001
SIRS/sepsis	WBC < 6 × 10^3^/μL	0.131	*p* < 0.05
Surgical site complications	BCS ≤ 2	0.071	*p* < 0.05
Major electrolyte imbalance	CI ≥ 2	11.783	*p* < 0.05
Major electrolyte imbalance	Postoperative use of vasopressors	0.258	*p* < 0.05
Postoperative hypotension requiring use of vasopressors	Tachycardia at admission	13.998	*p* < 0.001
Postoperative hypotension requiring use of vasopressors	SI > 1 at admission	13.998	*p* < 0.001
Postoperative hypotension requiring use of vasopressors	Intraoperative hypotension	0.081	*p* < 0.01
Postoperative hypotension requiring use of vasopressors	Perioperative use of blood product	0.137	*p* < 0.001

Variables significantly associated (*p* < 0.05) with postoperative complications in the multiple logistic regression analysis. SIRS = systemic inflammatory response syndrome; BCS = body condition score; CI = comorbidity index; SI = shock index; WBC =white blood cells; Hb = haemoglobin.

## Data Availability

The data presented in this study are available in this article.
